# A National Survey of Transplant Social Workers on Social Support Assessment and Intervention Practices

**DOI:** 10.1177/15269248261481281

**Published:** 2026-08-30

**Authors:** Gloria Chen, Katie Newton, Heather Bruschwein, Michelle T. Jesse

**Affiliations:** 1 21976 Abdominal Transplant Center, Dell Seton Medical Center at The University of Texas, Austin, TX, USA; 2 21645 Department of Transplantation, Baystate Medical Center, Springfield, MA, USA; 3 12349 Department of Psychiatry and Neurobehavioral Sciences, University of Virginia School of Medicine, Charlottesville, VA, USA; 4 5325 Consultation-Liaison Behavioral Medicine, Wellstar Regional Healthcare System, Marietta, GA, USA

**Keywords:** Transplant social work, psychosocial assessment, social support assessment, social support interventions, transplant psychosocial risk

## Abstract

**Introduction:**

Transplant candidates are assessed for caregiver support adequacy by transplant social workers as required by U.S. federal regulations. Research to date does not account nor adjust for interventions performed by transplant social workers to mitigate social support risk concerns on outcomes.

**Research Question:**

The objective of this study was to describe the social support assessment and interventions conducted by transplant social workers during the pretransplant evaluation process.

**Methods:**

A cross-sectional survey of transplant social workers was conducted through the Society for Transplant Social Workers. The survey inquired about social support assessment domains, requirements, and interventions to mitigate risk concerns.

**Results:**

A total of 141 transplant social workers participated. Majority were White (*n* = 124, 87.9%), female (*n* = 128, 90.8%) and held licensure at the highest level (*n* = 118, 83.7%). All organ groups and U.S. Organ Procurement Transplant Network regions were represented. The majority (*n* = 100, 70.9%) reported using a standard assessment tool. All reported multiple assessment domains within their social support assessment though with variability in overlapping domains. All participants reported conducting interventions to mobilize social support with notable overlap in interventions.

**Conclusion:**

Transplant social workers perform social support assessment and interventions to mitigate social support concerns. Research to date does not adequately capture nor adjust for interventions by transplant social workers to mitigate risk and facilitate transplant preparedness among potential transplant candidates. Future research should explore the effectiveness of transplant social work interventions to form guidelines for transplant social work practice.

## Introduction

Social support encompasses the informal caregivers and care partners who perform an essential role throughout the transplant process.^1^ The use of social support as a criterion for transplant has been widely debated^2^ because evidence linking social support to transplant outcomes, including medical adherence, graft survival, and patient survival, remains inconsistent.^3-5^ Nevertheless, social support has been associated with improved quality of life and illness perception among transplant patients.^6,7^

The Centers for Medicare and Medicaid Services (CMS) require transplant programs to maintain multidisciplinary teams that include Transplant Social Workers (TSWs).^8^ Federal regulations further mandate that all transplant candidates undergo a comprehensive psychosocial assessment, including an evaluation of the adequacy of their social support.^8^ TSWs are primarily responsible for conducting these psychosocial assessments and, in accordance with transplant center policies, evaluating whether candidates meet psychosocial criteria for listing. However, federal regulations do not prescribe specific psychosocial assessment methods or listing criteria. Instead, individual transplant centers determine how psychosocial factors are assessed and what level of psychosocial risk is acceptable for listing. Consequently, substantial variation exists across transplant centers in both psychosocial evaluation practices and transplant eligibility criteria.^9-11^

Despite federal mandates for psychosocial assessments, no universally accepted assessment tool or standardized social support listing criteria exist across transplant programs. Several instruments have been developed to guide psychosocial evaluation, with the Stanford Integrated Psychosocial Assessment for Transplantation (SIPAT) emerging as one of the more widely used tools.^9,12,13^ The SIPAT provides a semistandardized framework for assessing candidate risk across multiple psychosocial domains, including social support, and offers guidance regarding transplant candidacy based on cumulative risk scores. However, the SIPAT and other similar instruments^10^ remain heavily dependent on clinician judgment and have not been extensively validated against established psychometric measures. Existing validation studies primarily examine post-transplant outcomes among recipients using retrospective or case-control designs, introducing substantial selection bias by excluding candidates who were denied listing.^14^ This limitation is particularly relevant to debates surrounding social support as a transplant eligibility criterion. Although centers may attempt to address social support deficits, candidates unable to engage with or benefit from these interventions are often not listed and therefore are not represented in outcome studies. Consequently, the evidence base informing social support requirements is derived primarily from patients who successfully progressed through the evaluation process. In the absence of standardized assessment methods and listing criteria, social support requirements have been criticized as inconsistently defined and applied across transplant centers, potentially contributing to racial, ethnic, and socioeconomic disparities in access to transplantation.^2,10,15^

An additional limitation of the current literature is that psychosocial evaluation is often conceptualized as a single assessment rather than an ongoing clinical process. In practice, TSWs frequently conduct follow-up assessments and implement interventions aimed at strengthening social support, addressing identified barriers, and helping candidates meet transplant center requirements. Yet, these interventional efforts are rarely captured in empirical studies or considered in discussions regarding social support. One survey of transplant psychosocial providers found that only 30% reported using a formal validated tool to assess social support, while 44% reported always attempting to help patients strengthen inadequate social support.^9^ However, the sample included multiple provider disciplines and did not exclusively examine TSWs, who are most directly responsible for conducting psychosocial assessments and implementing psychosocial interventions.^9^ Therefore, given the limited understanding of how TSWs assess social support and intervene to address social support barriers, the purpose of this study was to describe the social support assessments and interventions conducted by TSWs during the pretransplant evaluation process.

## Design/Methods

### Design

A descriptive cross-sectional survey was conducted with TSWs. The study was approved by the Institutional Review Board of a large public academic institution (IRB #301072). This research study was conducted exclusively online.

### Population

TSW professionals within the United States who were identified as members of the Society for Transplant Social Workers (STSW) from May 2024 to June 2024. According to the STSW, TSWs are employed across all solid organ transplant patient populations, with the most found in kidney transplant (48%).^16^ Approximately 51% of TSWs hold a TSW credential, and approximately 27% of TSWs have between 6 and 10 years of TSW experience.^16^ Participants who consented to the study were asked to complete an online survey. Responses were anonymous. At the end of the survey, participants could indicate interest in participating in a focus group about the survey topics and submit contact information separately from their survey responses.

### Sampling

Participants were recruited through convenience sampling. Eligible participants were TSWs and members of the Society for Transplant Social Workers (STSW). Study participants were recruited through STSW. The survey was shared 3 times with the STSW membership from May to June 2024. Survey responses were collected via an anonymous link to preserve respondent confidentiality.

### Data Collection

Survey development was completed by the research team comprised of TSWs and health psychologists with transplant experience. The survey was tested with other transplant professionals including 2 TSWs, a transplant nurse, and a transplant surgeon. The survey was revised following feedback received from testing. The final 42-item survey included questions about demographics, the routine practices of transplant social support assessment, requirements, and interventions conducted intended to strengthen social support for transplant listing. Survey dissemination of STSW membership was reviewed and approved by the STSW before data collection.

### Data Analysis

Exploratory analyses were conducted examining participant responses. Analyses were largely frequencies and/or descriptive in nature. Additional subgroup analyses comparing responses across TSW personal and professional characteristics were not conducted because the distribution of respondents across numerous categories resulted in small subgroup sizes, limiting the interpretability of statistical comparisons. However, crosstab analyses were conducted across routine practices of transplant social support assessments and interventions conducted intended to strengthen social support for transplant listing. This was explored to further identify domains of common practice and determine opportunities for greater consistency across TSWs nationally.

## Results

A total of 141 (27.3% response rate) TSWs participated in this study (Table 1). Most participants were female (90.8%) and identified as White (87.9%). All organ groups and U.S. Organ Procurement Transplant Network (OPTN) regions were represented. While the average amount of years in practice as a social worker was 17.59 years, only 7.71 years were reported on average as practicing as a TSW. Most of the participants held a master's level social work degree (97.9%) and were licensed at the highest level within their jurisdiction (83.7%). Notably, almost exactly half held no transplant specialty social work certification. Participants reported conducting, on average, 100.4 transplant evaluations/year within an allotted 74.7 min/initial evaluation.

**Table 1.** Transplant Social Work Characteristics.

Variable	N (%)
Gender	
Female	128 (90.8%)
Male	13 (9.2%)
Race	
Black/African American	5 (3.5%)
Asian	4 (2.8%)
White	124 (87.9%)
Multiracial	5 (3.5%)
Other/Missing	3 (2.1%)
Hispanic/Latino Ethnicity	14 (7.5%)
Highest Degree	
Master's Degree	138 (97.9%)
Doctoral Degree	3 (2.1%)
Licensure Status	
Preliminary Licensure Status	23 (16.3%)
Licensure at the Highest level	118 (83.7%)
Specialty Social Work Certification	
CCTSW	54 (38.3%)
CCSW-MCS	4 (2.8%)
CCTSW-MCS	12 (8.5%)
No Certification	71 (50.4%)
Organ Specialization	
Kidney	33 (23.4%)
Heart*	29 (20.6%)
Liver	20 (14.2%)
Lung	10 (7.1%)
Combination of multiple organs	49 (34.8%)
OPTN Region	
Region 1	7 (5.0%)
Region 2	10 (7.1%)
Region 3	12 (8.5%)
Region 4	12 (8.5%)
Region 5	22 (15.6%)
Region 6	7 (5.0%)
Region 7	18 (12.8%)
Region 8	12 (8.5%)
Region 9	5 (3.5%)
Region 10	13 (9.2%)
Region 11	23 (16.3%)
Administrative time to prepare for an initial transplant evaluation– Yes	94 (66.7%)
	Mean (SD)
Years practicing as a social worker	17.59 (10.8)
Years practicing as a transplant social worker	7.71 (7.1)
Hours worked per week	40.44 (5.3)
Approximate number of transplant evaluations conducted per year at center	200.9 (220.6)
Approximate number of transplant evaluations conducted by social worker per year**	100.4 (167.4)
Time allotted by transplant center to conduct initial evaluation (in minutes)	74.7 (43.6)

*Heart includes mechanical circulatory support/left-ventricular assist devices.

**Team composition may impact reported number.

### Social Support Assessment

One hundred participants (70.9%) reported using a standardized assessment tool (eg, SIPAT, center-developed instrument) that is routinely administered to transplant candidates. Although all participants reported assessing multiple aspects of social support, there was variation in the specific domains included in their assessments (Table 2). The most frequently assessed domains were identifying the patient's social support network (99.3%) and evaluating caregiver availability to provide social support (97.2%). The domains most commonly assessed together were identifying the patient's social support network and evaluating caregiver availability, with 96.5% of participants reporting inclusion of both domains. Excluding the “Other” category, the least frequently assessed domains were collecting corroborative information to confirm caregiver(s) adequacy and identifying patient-specific medical needs that may require an increased level or certain type of support post-transplant, each reported by 58.2% of participants (Table 2). These findings suggest broad agreement among TSWs regarding the importance of identifying support networks and caregiver availability, but greater variability in how TSWs evaluate the quality and sufficiency of available support to meet transplant-related needs.

**Table 2.** Frequency of Transplant Social Support Assessment Elements and Overlapping Domains.

	N (%)	2.	3.	4.	5.	6.	7.	8.	9.	10.	11.
1. Identify the patient's social support network.	140 (99.3%)	96.50%	90.10%	83.00%	85.10%	74.50%	67.40%	94.30%	78.00%	77.30%	80.90%
2. Assess caregiver(s) availability to provide social support.	137 (97.2%)		90.80%	81.60%	84.40%	73.80%	66.70%	93.60%	77.30%	77.30%	80.90%
3. Assess caregiver(s) ability to provide social support.	128 (90.8%)			78.00%	80.90%	71.60%	66.00%	87.90%	73.8%	73.80%	78.00%
4. Assess caregiver(s) willingness to provide social support.	118 (83.7%)				78.00%	66.00%	61.00%	80.90%	68.80%	69.50%	70.90%
5. Assess patient and caregiver(s) relationship stability.	121 (85.8%)					69.50%	64.50%	82.30%	70.90%	72.30%	75.90%
6. Collect corroborative information to confirm caregiver(s) adequacy.	105 (74.5%)						58.20%	72.30%	65.20%	63.80%	67.40%
7. Identify unique patient medical needs that may require an increased level or certain type of support post-transplant.	96 (68.1%)							66.00%	59.60%	59.60%	61.00%
8. Identify source of transportation for all post-transplant appointments and other transportation needs.	134 (95.0%)								75.90%	75.90%	79.40%
9. Assess other sources of support resources in place including those paid for by insurance or out of pocket.	110 (78.0%)									69.50%	68.80%
10. Assess patient's financial means to pay for needed services post-transplant that are not covered by insurance.	110 (78.0%)										70.20%
11. Assess the patient's ability to manage medical and personal needs without a caregiver or other support plan.	115 (81.6%)										

Note: The “Other” category was omitted due to limited responses (frequency endorsed n = 6 or 4.3%, percent of overlap ranged from 2.8% to 4.3%). This table illustrates both the frequency of initial item endorsement (as reported by N (%)) and the overlap of TSW assessment domains that were reported by participants in this survey study. For example, 99.3% of respondents endorsed 1. Identify the patient's social support network, 97.2% of respondents endorsed 2. Assess caregiver(s) availability to provide social support, and 96.50% of respondents endorsed BOTH 1. Identify the patient's social support network AND 2. Assess caregiver(s) availability to provide social support.

### Social Support Requirements

Participants reported the social support requirements for transplant listing at their centers. Most participants (90.7%) reported that their transplant center requires a minimum number of supports (≥1) to meet listing criteria. Of the participants who reported that their transplant center requires a social support plan before transplant listing, the majority (83.6%) reported not ever making any exception to their listing criteria in their pretransplant assessment of the patient if that patient did not have a social support plan in place. However, notably, 20.6% reported their center has made exceptions to the center's listing criteria and 9.6% of participants reported having advocated for a patient to be listed for transplant without a social support plan. Remarkably, 60.3% of participants reported that their transplant center has listed patients despite TSW recommendation against listing due to inadequate social support.

Participants also reported the social support requirements included as a part of their assessments (Table 3). While most of the routine requirements identified were not required of all caregivers, most routine requirements were seen as essential for a patient's primary caregiver to complete, including attending transplant evaluation (63.8%) and post-transplant clinic appointments (73.8%) (Table 3). Furthermore, most TSWs reported that their transplant programs required the primary caregiver to provide 24/7 care to patients after transplant for a certain period (60.3%).

**Table 3.** Social Support Requirements.

	Required of All Social Support, N (%)	Required of the Primary Social Support, N (%)	Not Required, N (%)
Attend transplant evaluation appointment(s) with the patient	12 (8.5%)	90 (63.8%)	39 (27.7%)
Be available for evaluation via phone, if not available in person	48 (34.0%)	56 (39.7%)	36 (25.5%)
Attend family meeting for social support evaluation	18 (12.8%)	61 (43.3%)	60 (42.6%)
Attend transplant education appointment(s)	27 (19.1%)	82 (58.2%)	31 (22.0%)
Provide 24/7 care for patients after transplant for a period of time (eg, weeks)	31 (22.0%)	85 (60.3%)	25 (17.7%)
Provide care to the transplant recipient for a certain length of time, not specified to 24/7	34 (24.1%)	69 (48.9%)	35 (24.8%)
Attend post-transplant clinic appointments	17 (12.1%)	104 (73.8%)	19 (13.5%)
Provide meals and groceries	31 (22.0%)	69 (48.9%)	40 (28.4%)
Perform household chores (eg, laundry, cleaning)	32 (22.7%)	72 (51.5%)	36 (25.5%)
Manage medications	30 (21.3%)	88 (62.4%)	22 (15.6%)
Schedule appointments	21 (14.9%)	72 (51.1%)	45 (31.9%)
Provide transportation	38 (27.0%)	93 (66.0%)	10 (7.1%)

*Rows that do not equal 100% are due to nonresponses/missing data.

### Social Support Interventions

All TSWs routinely conduct interventions to mobilize social support and assist patients in meeting minimum listing criteria (Table 4). Most participants (93.6%) reported using multiple (≥2) interventions. These interventions commonly occur across several stages of the evaluation process, from initial psychosocial assessment through ongoing follow-up. More than half of participants (55.4%) reported contacting patients 2 to 5 times after the initial assessment to address identified psychosocial concerns. Almost all participants (97.2%) reported working with patients to identify potential social support, such as by strategizing how to approach individuals who could serve in a support role. Additional frequencies of reported support-mobilization efforts are presented in Table 4. Although participants reported using a variety of interventions, certain approaches were commonly used together. The most frequently co-occurring interventions were working with patients to identify potential social support and providing education sessions to social support people (78.0%). Excluding the “Other” category, the least frequently co-occurring interventions were facilitating social support meetings and collaborating with community-based care providers to assist in developing a social support plan (42.6%) (Table 4). These findings suggest broad use of multiple strategies to strengthen social support, with greater emphasis on patient-centered education and support identification than on multidisciplinary or community-based interventions.

**Table 4.** Frequency of Transplant Social Support Interventions and Overlapping Domains.

	N (%)	2.	3.	4.	5.
1. Work with patients to identify potential social support	137 (97.2%)	67.40%	78.00%	56.00%	56.00%
2. Facilitate social support meetings	98 (69.5%)		62.40%	45.40%	42.60%
3. Perform education sessions with social support	113 (80.1%)			48.90%	48.20%
4. Assess financial cost for services and make referral for supportive resources	81 (57.4%)				44.00%
5. Collaborate with other care providers in the community the patient is connected to for assistance in identifying social support plan	80 (56.7%)				

Note: The “Other” category was omitted due to limited responses (frequency endorsed n = 4 or 2.8%, percent of overlap ranged from 0.7% to 2.8%). This table illustrates both the frequency of initial item endorsement (as reported by N (%)) and the overlap of TSW intervention domains that were reported by participants in this survey study. For example, 97.2% of respondents endorsed 1. Work with patients to identify potential social support, 69.5% of respondents endorsed 2. Facilitate social support meetings, and 67.4% of respondents endorsed BOTH 1. Work with patients to identify potential social support AND 2. Facilitate social support meetings.

## Discussion

The present study was a cross-sectional survey describing social support assessment and interventions conducted by TSWs across the United States. A total of 141 TSWs participated, and most reported using a standardized social support assessment tool to evaluate routine social support requirements. Although CMS regulations require TSWs to assess social support adequacy for potential transplant candidates, 60.3% of respondents reported that their transplant center had listed patients whom the TSW had assessed as lacking an adequate social support plan. In addition, all respondents reported implementing interventions to mobilize social support, with substantial overlap in the strategies used across TSWs.

The high rate of standardized assessment tool use is consistent with broader trends toward structured psychosocial evaluation.^12,13^ Several instruments have been developed to assess psychosocial candidacy more systematically among transplant candidates.^10^ However, inconsistent evidence linking psychosocial assessment findings to transplant outcomes raises questions about how these assessments should be used in transplant candidacy determinations and may contribute to equity concerns.^10,17^ Standardized assessments may also inadvertently exacerbate disparities if they do not adequately account for individual patient contexts, cultural factors, and evolving social norms. For example, although most transplant programs require candidates to identify at least one primary caregiver, culturally specific models of family and community support may involve different configurations of caregiving that are equally effective. Rigid interpretations of caregiver minimums may inadvertently disadvantage candidates whose cultural norms emphasize collective or distributed caregiving rather than a single designated individual. Guidelines that promote culturally responsive assessment practices, along with training to reduce implicit biases in tool administration, may help ensure that these instruments serve the diverse population of transplant candidates more equitably.^17^

The finding that most respondents (60.3%) reported patients were listed despite TSW recommendation against listing due to inadequate social support raises important clinical and ethical considerations. These findings suggest that psychosocial concerns identified by TSWs may at times be weighed differently from medical considerations during multidisciplinary listing discussions. Although psychosocial and medical providers share the goal of achieving successful transplant outcomes, they may approach transplant candidacy from different professional perspectives. TSWs are responsible for assessing whether patients have sufficient support to manage the practical, emotional, and logistical demands of transplantation and post-transplant care, whereas medical providers may place greater emphasis on the potential clinical benefits of transplantation and the risks associated with delaying or denying access to transplant. Differences in how “adequate” social support is defined may be further compounded by the inconsistent evidence linking social support to transplant outcomes.^17^ This interpretation is consistent with findings from a national survey of transplant clinicians demonstrating that psychosocial and medical providers often differ in how they weigh social support, adherence concerns, and expected post-transplant outcomes when evaluating transplant candidates.^18^ Collectively, these findings highlight the need for research examining how psychosocial recommendations are incorporated into multidisciplinary decision-making and how differing professional perspectives influence transplant candidacy determinations.

Beyond assessing social support, all respondents reported conducting interventions to mobilize social support, and most reported using multiple (≥2) interventions (93.6%), underscoring the active role TSWs play in facilitating transplant readiness. Research examining the relationship between social support and transplant outcomes has generally treated social support as a patient characteristic without accounting for the interventions TSWs routinely implement to address identified support deficits and strengthen support networks. Consequently, the impact of social support on transplant outcomes may be difficult to interpret without considering efforts to mitigate psychosocial risk before transplantation. The substantial overlap in intervention strategies observed in this study suggests that TSWs are already engaged in informal standardization of care, even in the absence of formal protocols or guidelines. Few studies have captured psychosocial risk indicators among transplant candidates^19^ or the interventions used to address those risks and facilitate transplant preparedness. This gap in literature obscures the central role TSWs play on the multidisciplinary transplant team and limits understanding of how psychosocial interventions may influence transplant access and outcomes.

These findings also point to an opportunity for professional organizations to develop guidelines for social support assessment and interventions and promote more uniform delivery and practice. The Society for Transplant Social Workers is well positioned to lead efforts to build consensus around evidence-informed assessment practices and intervention strategies that promote consistency across TSWs and transplant centers. Additional training in social support assessment and intervention may further support standardization in practice. Clinician resource constraints remain an important consideration, and recent changes in the 2025 Medicare Physician Fee Schedule, which allow reimbursement for caregiver training, may provide transplant programs with new opportunities to strengthen caregiving support services.^20^ Lessons from oncology programs that have implemented these reimbursement codes could help inform similar efforts within transplantation.^20^ Increased financial support for caregiver training and related services may help expand psychosocial resources and support sustainable care delivery for transplant candidates.

This study has several limitations. Participants were predominantly White and female, which may limit the generalizability of the findings and the representation of diverse perspectives, particularly regarding issues of equity and disparities in transplantation. Although the sample included representation from all U.S. OPTN regions and reflected prior characterizations of the TSW workforce,^21^ future studies should seek to include more diverse participant samples. As a self-reported survey, the study is also subject to response and recall bias, and reported practices may differ from actual clinical behavior. Additionally, the survey was distributed exclusively to STSW members using a convenience sampling approach and achieved a 27.3% response rate, introducing the possibility of selection and nonresponse bias. STSW members and survey respondents may differ from nonmembers and nonrespondents in their professional engagement and clinical practices. Additionally, because the survey was distributed through a professional society membership list via an anonymous link to preserve respondent confidentiality, we were unable to verify duplicate responses or determine whether any eligible survey developers participated. Finally, although respondents reported the organ transplant populations they served, subgroup analyses by organ specialty were not conducted because many participants practiced across multiple organ programs and several organ-specific subgroups contained small sample sizes, resulting in overlap and sparse data that limited meaningful subgroup comparisons. Future studies should examine whether assessment tools and assessment practices differ across organ transplant specialties.

Future research should explore the effectiveness of TSW intervention strategies for addressing social support limitations among transplant candidates. Prior studies have yielded inconsistent findings regarding the relationship between social support and transplant outcomes and generally have not accounted for the interventions TSWs implement during the pretransplant period.^3^ Existing literature has also inadequately addressed the complex psychosocial factors that facilitate or hinder patients’ ability to engage in these interventions and progress toward listing. Additional research is needed to better understand the role and experience of TSWs and the impact of their intervention efforts on transplant candidacy decisions.

## Conclusion

This study examined the social support assessment and intervention practices among TSWs during the transplant evaluation process. Most participants reported using standardized assessment tools and implementing interventions to address social support concerns, with substantial overlap observed across assessment and intervention practices. These findings suggest emerging consistency in TSW approaches to evaluating and mobilizing social support and may inform the development of consensus guidelines to promote more standardized practice. Future research should evaluate the effectiveness of TSW intervention strategies and further examine the role of TSWs in addressing social support barriers and facilitating transplant candidacy. By incorporating TSW perspectives, this study provides important insight into the assessment and intervention practices used by TSWs to support transplant readiness.

## Abbreviations

CMS: Centers for Medicare and Medicaid Services
TSWs: Transplant Social Workers
STSW: Society for Transplant Social Workers
